# What Does It Mean?

**Published:** 1901-11

**Authors:** 


### WHAT DOES IT MEAN?

From time to time this journal has been the recipient of various curious documents from "St. Luke's Hospital of Niles, Michigan." They were, as received, regularly consigned to the waste-basket. It seems, however, that one at least does not feel satisfied,—Dr. E. O.

Kinsman, of Cambridge, Mass.,—and he sends the following energetic disclaimer against the unauthorized use of his name as the head of the subjoined circular.

While it has been, doubtless, very annoying to Dr. Kinsman to be placed in this position, those who know him and his long association with the best in New England would never think that knowingly he would consent to have his name connected with any organization forced to resort to such peculiar methods.

This whole business of St. Luke's Hospital seems to require explanation. It is possible some of our correspondents may be able to furnish the necessary information and answer the query, Why a hospital organized for a worthy purpose should find it necessary to resort to such questionable methods?

" Niles, Mich., August 2, 1901.

" Dear Doctor, We enclose you herewith some of our literature for your careful perusal, together with a Dentists' Application blank for you to fill out and return to us, should you wish to join our Dental staff.

" The many advantages, privileges, and financial benefits to be gained by your joining us are briefly and partially told as follows,—viz.:

" 1. We issue, in addition to our certificate, a neat lithograph pocket Membership Ticket, which we believe, if displayed judiciously, will pay for your certificate many times over during the course of a year. Should you want only our pocket Membership Ticket alone, it will cost you $2.00; otherwise, it goes free with the Certificate of Membership.

" 2. We have just received a very costly and ornamental Red Cross solid gold button from the wholesale jewellers, lettered in circular form: ' Staff St. Luke's Hospital,' which goes free with our new membership; or, if ordered alone, $2.00.

" 3. We will pay you a commission of twenty-five per cent. in cash for all surgical operations, and ten per cent. on all medical cases you may send to our hospital for treatment.

" 4. Should you wish to consult us at any time regarding difficult cases, we will freely give you whatever assistance and advice we can, and will make microscopical analysis of specimens sent us free of charge.

" 5. We charge nothing for nursing patients day or night, as part of the expense is taken from our Nursing Fund. We do charge, however, for board and rooms ranging from $1.50 to $2.00 per day, according to location selected by the patient.

" 6. After you have ordered and paid for the certificate of membership, *either in English or Latin*, should you so desire it, and will send us a list of names, not exceeding twelve, including your local newspapers, we will write an individual letter recommending you to each one of them. Of course these letters of endorsement are optional for you to accept or reject, whichever you see fit.

"Now Doctor, after considering all these strong features, we would ask, with all fairness, do you not consider it to your financial interests to have your appointment confirmed? Kindly let us hear from you as soon as possible, and greatly favor,

"Fraternally yours,
"ARTHUR C. PROBERT, M.D., D.D.S.,
"*President.*"

TO THE DENTAL PROFESSION OF THE UNITED STATES:

Be it known that I, Edgar O. Kinsman, D.D.S., of Cambridge, Mass., Secretary of the Massachusetts Dental Society and the Northeastern Dental Association, do hereby declare that the use of my name on the letter-heads of the St. Luke's Hospital of Niles, Michigan, is without my knowledge and consent. I declare it to be an unwarranted use of the same, and make this public declaration to set myself right before the profession and maintain my honor as an officer of the above-named societies.

(Signed) EDGAR O. KINSMAN, D.D.S.

CAMBRIDGE, MASS., September 21, 1901.

Commonwealth of Massachusetts } Suffolk, *s.s.*

BOSTON, September 24, 1901.

Personally appeared before me the above-named Edgar O. Kinsman, and made oath to the truth of the above statement subscribed to by him.

Before me,
WALDO E. BOARDMAN,
*Notary Public.*

